# Agency, Criminogenic Risk and Needs, and Recidivism: A Prospective Longitudinal Study Including 14,000 Adult Justice-involved Individuals

**DOI:** 10.1177/0306624X251349530

**Published:** 2025-07-08

**Authors:** Patrick Lussier, Pagnol Landry Kouassi, Julien Frechette

**Affiliations:** 1 School of Social Work and Criminology, Université Laval, Quebec City, Canada

**Keywords:** recidivism, risk assessment, corrections, longitudinal study, community reentry

## Abstract

This study examines the role and importance of agency, defined as the ability to recognize personal issues and motivation to change. More specifically, the study aims to explore whether agency can help overcome criminogenic risk and needs in the context of community re-entry among justice-involved individuals. Based on a sample of 14,000 adult males sentenced to probation or incarceration, a series of survival analyses (e.g., Cox proportional hazards) were used to investigate the association between criminogenic risk and needs and agency-related indicators in relation to recidivism. The findings underscore the importance of criminogenic risks and needs while emphasizing the role of motivation to change as a possible moderator. Addressing criminogenic risk and needs while justice-involved individuals face numerous barriers and challenges make desistance from crime a long and difficult process, especially if interventions do not support agentic decisions and behaviors.

## Plain language summary

**Examining whether justice-involved individuals can overcome** **criminogenic risk and needs through agency and the motivation to change their life course**

Why was the study done? A significant proportion of justice-involved individuals return in the criminal justice for a new criminal offense. Several approaches have been proposed to break this cycle. Among those, correctional services have relied on the risk-need-responsivity which stresses the importance of targeting criminogenic risk and needs through treatment and intervention. The role and importance of agency—the motivation to change their life course—as a key component of change and desistance from crime, has been neglected. What did the researchers do? The research team examined how important agency is by examining the community reentry of 14,000 justice-involved individuals and criminal recidivism over a maximum of an 8-year period. What did the researchers find? The research shows that, as expected, justice-involved individuals with more criminogenic risk and needs were more likely to recidivate. Individuals with very high risk and needs were 13 times more likely to recidivate than the very low risk and needs group. The findings did show that individuals who recognized their personal issues and were motivated to change were less likely to recidivate, especially among the higher risk and needs group. What do the findings mean? The findings reiterate that while agency is not sufficient for justice-involved individuals to change and desist from crime, it is part of the process. Addressing criminogenic risk and needs, especially for individuals facing challenging life adversities, can make desistance a long and difficult process, especially if interventions do not support agentic decisions and behaviors.

In the context of rising crime rates throughout the 1970s and 1980s and following empirical observations that incarceration carried no deterrent effect (e.g., Gendreau et al., 1996; Loeffler & Nagin, 2022; E. C. McCuish et al., 2025), the study of criminal recidivism burgeoned, especially in Canada and the United States. Three research streams have offered different perspectives on justice-involved individuals and repeat offending, each with distinct implications for crime prevention. The first stream, heavily influenced by the criminal career paradigm, proposed a static view in which the propensity to commit crimes is relatively fixed and, therefore, offending is considered relatively predictable (e.g., A. Blumstein et al., 1986; Chaiken & Chaiken, 1984; Le Blanc & Fréchette, 1989; Petersilia, 1980). The second stream, presented through the risk-need-responsivity (RNR) principles of effective intervention (Andrews, Bonta, & Hoge, 1990), espoused a more dynamic view where recidivism can be prevented by focusing on evidence-based interventions targeting changeable dynamic risk factors (e.g., Caudy et al., 2013). This second stream prioritizes risk management strategies over prediction, tailoring interventions to individuals’ criminogenic risk and needs to facilitate community reentry and reintegration (e.g., Andrews et al., 2006; Lowenkamp et al., 2006). The third research stream, which has gained prominence since the early 21st century (Bersani & Doherty, 2018), focuses on desistance from crime. This stream focuses less on criminogenic needs and criminal justice interventions and more on the importance of agency, offenders’ strengths, and justice-involved individuals’ decision to change (e.g., Carlsson, 2016; Healy, 2013; Paternoster et al., 2015). This perspective posits that agentic actions and decisions are central to desistance from crime. The development of criminological thinking around prediction, rehabilitation, and desistance has often been framed as a competition among viewpoints, leading to frequent misrepresentation and misunderstandings. To advance the field of research, it is essential to contextualize these ideas beyond ideological frameworks and reasoning. More specifically, the present study examines justice-involved individuals’ agency and their ability to make their own life choices, act independently, and exert internal motivation to change. As a result, this study examines whether the concept of agency should be considered in relation to criminogenic risk and needs to better understand the recidivism process, with implications for service delivery and intervention practices. Drawing on data from a prospective longitudinal study with a large, representative sample of justice-involved individuals, this research integrates key ideas from the three research streams. Specifically, it investigates whether an individual’s agency—demonstrated through an awareness of their personal challenges and a motivation to change—can mitigate criminogenic risk and needs. In other words, this study takes an integrative approach, exploring whether individuals can overcome their criminogenic risk and needs through agency.

## Literature Review

The first research stream focused on identifying and predicting high-risk offending among justice-involved individuals through various means. There is a long tradition of research on the prediction of recidivism, which includes the influential work of Burgess (1928) as well as Sheldon and Eleanor Glueck (Glueck & Glueck, 1959), which aimed to assist parole board decision-making. The development of risk prediction tables gained early recognition in mental health settings as researchers demonstrated its superiority over unstructured clinical judgment (e.g., Meehl, 1954), a conclusion that has since been further supported by evidence (e.g., Ægisdóttir et al., 2006; Grove & Meehl, 1996). During the 1980s, this research stream received a significant boost from researchers invested in the development of the criminal career paradigm (A. Blumstein et al., 1986; DeLisi & Piquero, 2011; Farrington, 1990; Piquero et al., 2003). The criminal career paradigm emerged in a period of skepticism about the effectiveness of offenders’ treatment programs in corrections and the rise of anti-rehabilitation biases (e.g., Martinson, 1974; see also, Andrews et al., 1990; Palmer, 1975). Criminal career researchers recognized the presence of much heterogeneity in offending and contributed to the development of a criminal career paradigm aimed at identifying the number, prevalence, and shape of criminal careers, along with associated predictors (e.g., Barnett et al., 1987; Greenberg, 1991; Nagin et al., 1995). This research stream included the early identification of chronic and serious offenders, using mainly static, historical risk factors (e.g., criminal history; e.g., see Hester, 2019) which raised several concerns (e.g., S. D. Gottfredson & Gottfredson, 1994; LeBlanc & Loeber, 1998). This research agenda sparked a heated debate between criminal career researchers who sought a model for classifying criminal careers, while propensity theorists argued that such research was futile since offending was best understood as a continuum or propensity (e.g., Blumstein et al., 1988; M. Gottfredson & Hirschi, 1986). The debate was not settled on university campuses or at academic conferences but rather by the criminal justice system, which gradually changed its practices to incorporate actuarial risk assessment and prediction tables (e.g., Feeley & Simon, 1992). More specifically, the criminal justice system recognized the need for a classification system to guide decision-making, one that captured a continuum of recidivism risk probabilities. Today, actuarial risk assessment remains a cornerstone of criminal justice practices and influences both front-end and back-end decision making (e.g., Goel et al., 2021).

The second research stream emerged from a psychocriminology perspective emphasizing rehabilitation as a guiding principle of sentencing. While prediction tables were gaining popularity, especially in the U.S., a group of scholars challenged this trend (e.g., F. T. Cullen, 2005; Hollin, 1999). This group challenged the development of actuarial risk assessment tools that did not recognize offenders’ ability to change and the impact of effective interventions (e.g., Andrews, Zinger et al., 1990; Gendreau et al., 1996). This work culminated in the development of principles of service delivery in corrections, known as the RNR model. The RNR model emphasizes the importance of personal change, including the role of training and interventions for criminal justice professionals in facilitating that change. To capture this change, researchers argued that dynamic, changeable risk factors would need to be considered, especially those that are conceptually and empirically associated with recidivism. To promote change, the criminal justice system needed to tailor its interventions based on a person’s level of criminogenic risk and needs. To accomplish this, criminal justice professionals required training to promote cognitive and behavioral change (e.g., Dowden & Andrews, 2004). While endorsing some ideas of the first research stream by recognizing the value of actuarial risk assessment (e.g., Bonta, 2002), they departed from its prediction-focused objectives and instead stressed the importance of service delivery and interventions as key mechanisms for change. It has been argued that such interventions should be centered on eight criminogenic risk and needs, also known as the “C8” (e.g., Andrews et al., 2006). The influence of this research stream extends beyond the scholarship it produced to its impact on criminal justice policies and practices. This impact includes the development of assessment and classification tools, intervention approaches and programs, and professional skills to intervene with justice-involved individuals (e.g., Labrecque & Smith, 2017; Latessa et al., 2002). Because the second research stream emphasizes risk assessment to guide interventions, time and time again, it has been mistaken for a prediction-focused research stream. In fact, researchers from the second research stream have stressed the importance of looking beyond offenders’ risk and needs and instead emphasizing change and rehabilitation (e.g., Day et al., 2006).

The third research stream emerged from the recognition that desistance from crime is the norm and that valuable insights could be gained by examining the process by which individuals stop offending (e.g., Bushway et al., 2001; Kazemian, 2007). An age-graded theory of desistance by Laub and Sampson (2003) stressed the unpredictability of human behavior and the randomness of life events, which play a critical role in an individual’s decision to continue or stop offending. This perspective challenged classification and prediction models and associated practices. Initially, this perspective was criticized on several grounds for oversimplifying human lives, failing to explain well-known patterns of offending, and not properly contextualizing the process of change (e.g., Carlsson, 2012; Gadd & Farrall, 2004; E. C. McCuish, 2020; E. McCuish & Lussier, 2023). Notably, Laub and Sampson’s (2003) theory offered an ambiguous stance on the role of human agency and self-determination, as well as the extent to which individuals actively shape their own lives (Paternoster et al., 2015), ideas that were unambiguously presented as key concepts of a theory of desistance by others (e.g., Paternoster & Bachman, 2017). While entering prosocial roles such as marriage and employment were described as initiating the desistance process (Laub & Sampson, 2003), others stressed the importance of a cognitive transformation (e.g., Maruna, 2001), described by P. C. Giordano et al. (2002, p. 1000) as a “shift in the actor’s basic openness to change” for desistance to occur. Rather than focusing on individual challenges and criminogenic risk factors—the hallmark of the second research stream—desistance-led research emphasized individuals’ capacity for change by redefining their identity and life goals (e.g., Bachman et al., 2016).

This research stream evolved by focusing on the importance of human agency (e.g., Johnston et al., 2019; King, 2013a; Paternoster et al., 2015) which refers to individuals’ ability to make their own life choices, act independently, and exert internal motivation to change. The absence of agency can be observed in individuals’ inability to reflect on their own actions, as well as their tendency to minimize and rationalize their involvement in criminal activities (e.g., Liem & Richardson, 2014). However, whether changes in self-identity correspond to actual behavioral changes remains a subject of debate (Rocque et al., 2016). The retrospective nature of qualitative studies characterizing this stream of research makes it difficult to draw firm conclusions. While self-determination is critical, it has limitations, as change does not occur in a vacuum and is contingent on environmental and contextual factors (e.g., Grattet et al., 2011; Lussier & McCuish, 2016). Specifically, self-determination, agentic decisions and actions toward behavioral change may not be that important when reoffending is highly unlikely. Agency may also be insufficient under difficult life circumstances (e.g., P. C. Giordano et al., 2002; Maruna & Farrall, 2004). For agentic decisions to help consolidate individual changes and foster a new identity, justice-involved individuals may experience the “pains of desistance” as they struggle to access social roles and employment, which are critical for rebuilding their lives (e.g., Fredriksson & Gålnander, 2020; Halsey et al., 2016; Healy, 2017; Lussier & McCuish, 2016; Nugent & Schinkel, 2016).

### Aim of the Study

It is now well established that criminogenic risk and needs play a critical role in justice-involved individuals’ decision to reoffend. Despite its strengths and the supporting empirical evidence (see F. Cullen, 2013), the RNR model and principles, including the criminogenic risk and needs literature, do not provide a theoretical model of change. The RNR model includes key principles of effective service delivery in correctional settings, identifying criminogenic risk and needs as primary targets for intervention. It also stresses the idea that individual change can be achieved by criminal justice professionals using effective intervention skills (or core correctional practices) that are aligned with learning theory and principles to help promote change (e.g., Dowden & Andrews, 2004; Viglione & Labrecque, 2021). The RNR model, however, does not describe the process of change (e.g., see P. C. Giordano, 2022) and the context that shapes it. In fact, the RNR model tends to depict justice-involved individuals as passive actors receiving an intervention using social learning theory principles rather than playing an active role in shaping their lives as emphasized in desistance research. For example, such principles stress the type of interventions strategies that criminal justice professionals should use to bring about a change in justice-involved individuals (e.g., being firm but fair, reinforcing anticriminal attitudes, teaching concrete problem-solving skills, using open and warm communication; e.g., see Dowden & Andrews, 2004). This gap raises questions about the nature of change and the underlying processes affecting individuals with criminogenic risk and needs. To be clear, the goal of the current study is not to empirically test a model of change but rather to explore whether the desistance literature provides a theoretical template that could help expand RNR principles of service delivery in correctional settings. On the one hand, the developers of the RNR principles (e.g., Andrews & Bonta, 2010) did not specifically address the research on agency and desistance from crime, but they recognized the importance of justice-involved individuals’ responsivity to intervention, which refers to, among other things, individuals’ recognition of personal issues and motivation to change (Andrews, 2006). These two features, central to this study, capture at least some aspects of the concept of agency as described in the desistance literature. On the other hand, while recognizing the value of agency, F. T. Cullen (2017) expressed reservations about theoretical perspectives that focus solely on agency without accounting for criminogenic risk and needs as it suggests that, under such assumptions, criminal behavior is depicted only as a matter of choice. Consequently, researchers such as Brezina (2020) argued for theoretical integration by examining when and under what conditions agency is most likely to occur (see also Piquero, 2020).

While the risk and needs principles have been the subject of an impressive number of studies, it remains unclear how the responsivity principle is applied by criminal justice practitioners as part of their interventions (e.g., Bonta et al., 2011; Finseth et al., 2022; Looman et al., 2005). For desistance-focus researchers, justice-involved individuals are responsible for their own change, constructing their lives through agentic decisions and actions. However, the social context surrounding an individual’s reentry into the community is also critical, as it should provide opportunities for change (e.g., Anderson, 2019; Youssef et al., 2016). Criminal justice professionals can assist justice-involved individuals in making these life changes through counseling and connecting them with community-based resources. This led researchers, such as Serin and Lloyd (2017) as well as Horan et al. (2020) to argue that these desistance-relevant factors such as agency should be integrated into interventions targeting criminogenic risk and needs. In this article, we examine whether justice-involved individuals’ agency can mitigate criminogenic risk and needs, regardless of their level of risk. While Carlsson (2016) emphasized that individuals must actively desire change and influence their life trajectories, P. Giordano (2017) and P. C. Giordano (2020) argued that recognizing personal issues and expressing motivation to change do not necessarily translate into behavioral change, as such change is contingent on external pressures and challenges. Therefore, we hypothesize that agency alone is not sufficient for some individuals, particularly those facing challenging life adversities. We test the hypothesis that some of these barriers stem from criminogenic risk and needs, as outlined by the C8 model (Andrews et al., 2006) using data from a large sample of 14,000 adult justice-involved individuals sentenced to either probation or incarceration and followed in the community for an average of 5 years. Criminogenic risk and needs were assessed using the LS/CMI, a risk and needs assessment instrument completed by criminal justice professionals during the intake assessment.

## Methodology

### Sample

The sample includes all individuals who were consecutively convicted and sentenced to at least 6-month probation and/or a custodial sentence (less than 2 years) between 2008 and 2011 in the province of Quebec, in Canada. This sample represents a quasi-population of all justice-involved individuals sentenced to probation or short-term incarceration during that period in the province of Quebec. The inclusion of both probation and custodial sentences allows for a more diverse sample while accounting for the relative impact of sentencing. The total sample consisted of a total of 15,717 individuals, of whom 89.1% were male. Given the low prevalence of women in the sample and to avoid potential confounding effects, this study focused exclusively on males (*n* = 14,000). Regarding the index offense, 30.5% of the sample had been convicted of a violent crime, 23.1% of a property crime, and 19.5% of a drug-related crime. Descriptive information about the sample is presented in Table 1.

**Table 1.** Descriptive Statistics About the Sample.

Variables	% (*n*)	Mean (*SD*)
Age		36.1 (12.1)
Sex		
Male	100.0 (14,000)	
Type of sentence		
Incarceration	40.7 (5,696)	
Probation	59.3 (8,304)	
Index crime		
Violent	30.5 (4,266)	
Property	23.1 (3,238)	
Drug-related	19.6 (2,742)	
Sexual	3.6 (500)	
Other offenses	23.2 (3,254)	
Number of prior adult convictions		6.8 (9.0)
LS/CMI total score		20.7 (9.3)
LS/CMI risk level		
Very low	3.9 (551)	
Low	12.4 (1,741)	
Moderate	30.0 (4,193)	
High	34.2 (4,793)	
Very high	19.4 (2,722)	
LS/CMI subscales (C8 factors)		
Criminal history		4.6 (2.4)
Procriminal attitude/orientation		1.3 (1.3)
Antisocial pattern		1.5 (1.2)
Companions		2.3 (1.2)
Alcohol/drug problems		3.4 (2.4)
Leisure/recreation		1.4 (0.7)
Education/employment		4.5 (2.9)
Family/marital		1.7 (1.2)
Potential barriers		
Homelessness (yes)	4.0 (566)	
Mental health problems (yes)	11.8 (1,651)	
Financial problems (yes)	37.3 (5,226)	
Agency		
Motivation for change (yes)	66.5 (9,306)	
Absence of denial/minimization (yes)	47.5 (6,656)	
Follow-up period (months)		61.4 (29.2)
General recidivism		
No	58.0 (8,122)	
Yes	42.0 (5,878)	

*Note.* Sample size is 14,000.

### Procedures

The current study is part of a larger study examining the community re-entry and reintegration issues (e.g., Frechette & Lussier, 2023; Lussier et al., 2025; Lussier & Frechette, 2022). Since the enactment of the *Loi sur le système correctionnel du Québec* in 2007, offender correctional records have been computerized and centralized by Quebec’s Ministry of Public Safety. For this study, a computer analyst from the Ministry of Public Safety extracted and pooled anonymized data from these records. During this process, each justice-involved individual was assigned a random ID number. The extraction process included information on risk and needs assessments, which were conducted as part of routine duties by criminal justice professionals rather than for research purposes. Additionally, computerized correctional records were analyzed to identify the nature of their index offense. A similar procedure was used to extract information about recidivism.

### Measures

#### Central Eight

The C8 was operationalized into criminogenic risk and need factors as part of the Level of Service risk assessment instrument family. For the purpose of this study, data were collected using the *Level of Service and Case Management Inventory* (LS/CMI, Andrews et al., 2004). Intake assessments and the LS/CMI were completed by trained criminal justice professionals with a university degree (e.g., criminology, social work). Given that Quebec is a predominantly French-speaking province, a French version of the LS/CMI was used (e.g., Guay, 2016). The psychometrics properties and associated issues of the LS/CMI have been previously examined and extensively discussed (e.g., Giguère & Lussier, 2016, 2017). As part of their duties, trained criminal justice professionals, are required to complete a risk and needs assessment. Completing the LS/CMI requires a rigorous investigation that considers numerous sources of information such as police and court data, prior correctional and institutional files, interview with justice-involved individuals, information from their family members, and information from other professionals. For this study, information stemming from the first section of the instrument allows measuring criminogenic risk and need factors, or the C8 (Andrews et al., 2004). The C8 includes eight subscales comprising a total of 43 items: criminal history (eight items); education/employment (nine items); family/marital (four items); leisure/recreation (two items); companions (four items); alcohol/drug problem (eight items); procriminal attitude/orientation (four items) and antisocial pattern (four items). The combination of C8 items, using a simple additive procedure, was calculated for each subscale and for all the subscales combined. The combination of all subscales is referred to as the risk score (Mean = 20.7; *SD* = 9.3; range = 0–43). Both the total risk/needs score and the score for each subscale was used in the current study. The higher the score, the higher the risk and needs, and by extension, the intensity of intervention and community supervision. The descriptive statistics for the criminogenic risk and need subscales are presented in Table 1. In total, 34.2% of the sample was designated as high-risk and 19.4% as very high-risk based on their LS/CMI total score. In contrast, 3.9% were designated as very low risk, and 12.4% as low risk.

#### Agency

In criminology, the concept of agency has primarily been discussed in relation to desistance, reflecting personal decision-making (see P. C. Giordano, 2020). From a life course perspective, agency has been described as the ability to exert control over and give direction to one’s life (Biesta & Tedder, 2007). In this conceptualization, personal agency implies that an individual has learned from past experiences and intends to act in ways that shape a future different from their past (e.g., Emirbayer & Mische, 1998). This view of agency aligns with Prochaska and Velicer (1997) transtheoretical model, which outlines the process by which individuals make agentic behavioral changes. While this study does not aim to test the transtheoretical model of change, it is believed that the indicators used tap into the early stages of change. As part of their professional duties, criminal justice professionals (e.g., social workers, criminologists^1^) are trained to assess justice-involved individuals’ openness to change. Two items of the LS/CMI reflect this assessment and these items were used as part of the current study. These dichotomous items (presence/absence) were coded by criminal justice professionals based on their assessment of the situation. The first item reflects whether justice-involved individuals tend to deny and/or minimize their personal issues. The second item reflects whether justice-involved individuals demonstrate a motivation for change. These two items align with Prochaska and Velicer’s (1997) precontemplation stage (e.g., no intention to act in the foreseeable future) and contemplation stage (e.g., intention to change) of behavioral change. Both items were coded to reflect the absence of denial/minimization about personal issues (coded as 1, as opposed to 0) and the presence of motivation to change (coded as 1, as opposed to 0). It is important to note that this study does not aim to examine the stability and change in agency during a specific sentence. Instead, the goal is to determine whether agency-related indicators as measured at the intake assessment can moderate criminogenic risk and needs.

#### Barriers

The current study statistically controls for potential barriers that could limit a person’s responsivity to interventions, their agentic decision-making, and their behavior toward desistance from crime. The presence of a history of mental health problems (dichotomously coded as present/absent) was based on a formal diagnosis made by a mental health professional. Three types of diagnoses were considered, that is, severe mental health problems (e.g., schizophrenia), personality disorders other than antisocial personality disorder^2^ (e.g., borderline personality disorder) and psychopathy. In total, 11.8% of the sample had a history of mental health problems as defined above. The presence of a history of homelessness (dichotomously coded as present/absent) was based on criminal justice professionals’ assessment of the person’s situation during the intake assessment. This issue was observed in 4% of the sample. Finally, financial problems and difficulties, which were present for 37.3% of the sample, were also statistically controlled for.

#### Criminal Recidivism

There are multiple ways to operationalize the concept of desistance, each with their strengths and limitations (e.g., Lussier et al., 2016). Information on recidivism was gathered for all individuals included in the sample. Information was gathered between March 2008 and February 2016 for a maximum follow-up period of 8 years. The follow-up period began at different times depending on the type of sentence. Community follow-up began immediately after sentencing for those serving a sentence in the community, whereas the follow-up began only when individuals who had received a prison sentence were released from custody. Note that the incarceration periods were short, lasting less than 2 years. In the context of this study, general recidivism refers to a new conviction for any criminal offense (0 = No; 1 = Yes) during the follow-up period, including probation and parole breach. On average, individuals were followed for 61 months, or about 5 years.

### Analytical Strategy

A series of Cox proportional hazard models (Cox, 1972) were estimated to determine the association between criminogenic risk and needs and recidivism while adjusting for offenders’ agency and exposure to potential barriers. This type of analysis has been repeatedly used in prior studies to test key assumptions stemming from the three streams of research (e.g., Kurlychek et al., 2012; Lussier & Frechette, 2022). Proportional hazard models are appropriate because they allow examining discrete events (i.e., criminal recidivism) while handling multiple covariates (e.g., Allison, 2010). Such statistical models also allow handling the timing of recidivism while statistically controlling for right-censoring, which is common for this type of data (e.g., Hester, 2019; Lussier & McCuish, 2016). Survival analyses are specifically designed to handle censored data without removing cases or creating biases, which is problematic in methods like logistic regression. Statistically controlling for the length of the follow-up period is critical, as all sample members returned to the community at some point, but some sooner than others. These analyses account for the possibility that, with a longer follow-up period, individuals who did not recidivate initially may do so later (e.g., see E. C. McCuish, 2020).

## Results

### Recidivism Rate and Survival Curves

**Figure 1.**
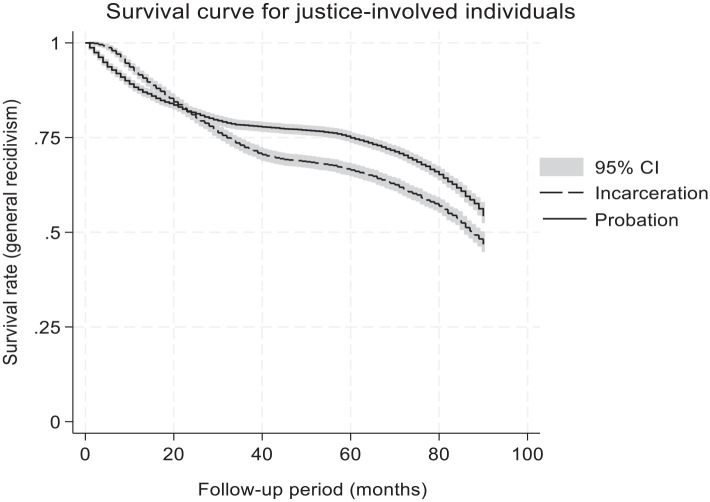
Survival curve of justice-involved individuals using a Kaplan-Meier estimator.

For the entire sample, the base rate of general recidivism was 42.0% (*n* = 5,884), meaning that they were convicted at least once for a new criminal offense during the study period. Given the relatively short follow-up period (i.e., about 5 years), the percentage of the sample with more than one reconviction was relatively small (12.7%). The survival curve was examined to assess the pace of recidivism events. This analysis was conducted until the 92nd month, after which less than 10% of the sample remained. When examining recidivism rates, the proportion of recidivism events was highest and steady for the first 2 years (about 5%–6% across 6-month periods), gradually dropping thereafter to about 3 to 4% in the third year to 1% in the fifth year. Furthermore, a higher percentage of individuals who served a custodial sentence recidivated (47%) than those sentenced to probation (38.5%). The survival curves by sentencing are presented in Figure 1 which were statistically different [*X*^2^(1) = 116.4, *p* < .001]. While some probationers were quick to recidivate, individuals who served a custodial sentence outpaced them after around the second year following their community re-entry.

**Figure 2.**
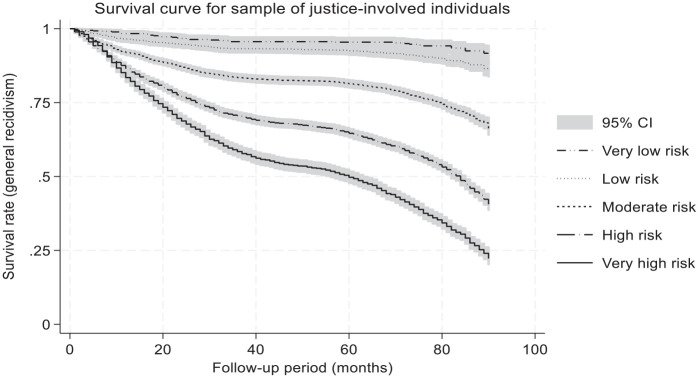
Survival curves of justice-involved individuals according to risk level using a Kaplan-Meier estimator.

Next, the survival curves were examined based on individuals’ criminogenic risk and needs level as determined by the LS/CMI scores. As shown in Figure 2, the survival curves were reflective of and consistent with individuals’ risk and needs level. For all groups, the risk of recidivism was highest during the first year, with yearly risk probabilities decreasing over time. The very low and low risk group showed the highest proportion of individuals surviving the period without a recidivism event. The first year showed the highest proportion of recidivism events, but these remained relatively rare (2% for very low; 4% for the low-risk group). The first year was also critical for the moderate risk (8% with a recidivism event), the high-risk (13%) and the very high risk (16%) groups. The LS/CMI risk and needs level provides useful information about the statistical probabilities of surviving a recidivism event during the follow-up period [*X*^2^(4) = 2402.2, *p* < .001] but recidivism was not a certainty for this sample even for the high and very high risk and needs groups.

**Figure 3.**
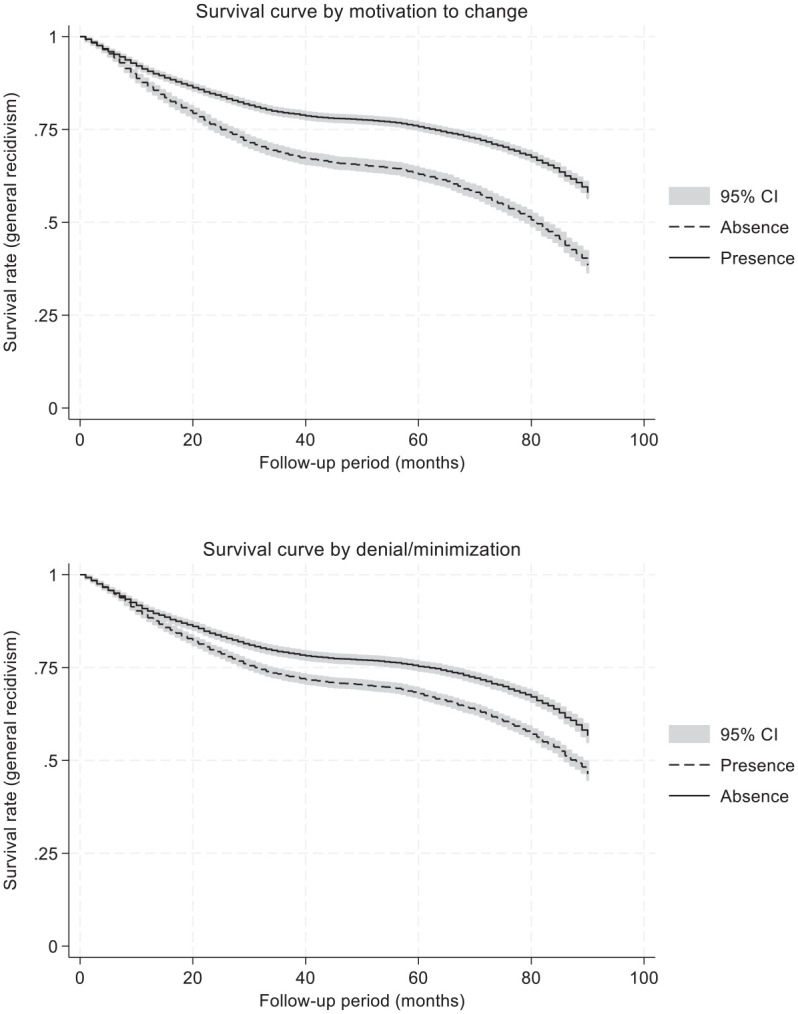
Survival curves of justice-involved individuals using a Kaplan-Meier estimator according to agency indicators.

Figure 3 show the survival curves of justice-involved individuals for two agency-relevant indicators. The findings highlight that individuals who did not exhibit denial and minimization toward their personal issues [*X*^2^(1) = 147.4, *p* < .001] and those who showed motivation to change [*X*^2^(1) = 421.1, *p* < .001] recidivated less and at a slower pace. The difference was somewhat more pronounced for the indicator reflecting individuals’ motivation to change compared to the one related to denial/minimization. More specifically, individuals who did not manifest a motivation to change during the intake assessment recidivated at a faster pace, especially during the first 30 months of the follow-up period. Note that these analyses do not simultaneously account for other covariates; therefore, a series of Cox regression analyses were conducted.

### Cox Proportional Hazards and Covariates’ Main Effects

Investigating covariates using Cox proportional hazard models was crucial, as most individuals did not experience recidivism during the follow-up period, although the risk probabilities did vary according to criminogenic risk and needs. Three Cox regression models were examined, and all three models were statistically significant (*p* < .001; see Table 2). The first model included the two agency indicators, while statistically controlling for age, type of sentence, the index crime as a violent offense, the total number of adult convictions as well as the three barrier indicators. This regression model revealed that, after controlling for these covariates, both agency indicators were statistically associated with recidivism. More specifically, recognizing personal issues was associated with an 11% reduction in recidivism rates while being motivated to change with a 25% reduction in risk. The model also showed that older individuals had a significantly lower risk of general recidivism, while those convicted of violent crimes and individuals with more prior adult convictions showed higher recidivism risk. That model also indicated that having experienced homelessness, mental health problems, and financial difficulties were associated with a significantly higher risk of recidivism.

**Table 2.** Cox Regression Analyses of Covariates of Recidivism.

	Model 1 (Baseline)	Model 2 (Risk level)	Model 3 (Criminogenic risk and needs)
Covariates	Hazard ratio (95% CI)	Hazard ratio (95% CI)	Hazard ratio (95% CI)
Age	0.97 [0.97, 0.97]***	0.98 [0.97, 0.97]***	0.98 [0.97, 0.98]***
Type of sentence (probation)	0.98 [0.93, 1.04]	1.28 [1.21, 1.35]***	1.41 [1.33, 1.50]***
Index crime (violence)	1.29 [1.22, 1.36]***	1.17 [1.11, 1.24]***	1.14 [1.08, 1.21]***
Number of prior adult convictions	1.03 [1.03, 1.04]***	1.02 [1.02, 1.03]***	1.02 [1.01, 1.02]***
Agency			
Motivation for change (presence)	0.75 [0.71, 0.79]***	0.97 [0.91, 1.03]	0.97 [0.91, 1.03]
Denial/minimization (absence)	0.89 [0.84, 0.94]***	1.04 [0.98, 1.10]	1.01 [0.95, 1.08]
Potential barriers			
Homelessness	1.28 [1.15, 1.42]***	1.21 [1.09, 1.35]***	1.16 [1.04, 1.29]***
Mental health problems	1.30 [1.21, 1.39]***	1.11 [1.03, 1.19]**	1.08 [1.00, 1.16]
Financial difficulties	1.19 [1.13, 1.26]***	0.99 [0.94, 1.04]	1.00 [0.95, 1.05]
LS/CMI risk-need level^a^			
Low	-	1.93 [1.36, 2.74]***	-
Moderate	-	4.71 [3.40, 6.55]***	-
High	-	9.37 [6.76, 13.00]***	-
Very high	-	13.16 [9.44, 18.35]***	-
LS/CMI subscales			
Criminal history	-	-	1.47 [1.41, 1.53]***
Procriminal attitude/orientation	-	-	1.04 [1.00, 1.08]*
Antisocial pattern	-	-	0.98 [0.94, 1.02]
Companions	-	-	1.07 [1.03, 1.11]***
Alcohol/drug problems	-	-	1.24 [1.21, 1.28]***
Leisure/recreation	-	-	1.08 [1.05, 1.12]***
Education/employment	-	-	1.13 [1.09, 1.17]***
Family/marital	-	-	1.08 [1.05, 1.12]***
Model fit *X*^2^(df), *p*-value	2,105.7 (9), *p* < .001	2,920.6 (13), *p* < .001	3,112.9 (17), *p* < .001

*Note*. Sample size is 14,000.

a The category of reference is very low group.

* *p* < .05. **p < .01. ***p < .001.

The second model includes all covariates from the first regression model, with the addition of the LS/CMI risk and needs level. This regression model allowed examining whether the two agency indicators remained associated with lower risk of recidivism while holding constant individuals’ overall level of criminogenic risk and needs. The findings indicate that as criminogenic risk and needs increase, the likelihood of general recidivism also rises. For instance, individuals in the moderate risk group were 4.7 times more likely to recidivate than those in the very low risk group. Similarly, individuals in the very high risk group were 13 times more likely to recidivate than those in the very low risk group. After incorporating the LS/CMI criminogenic risk and needs level, the second model showed that the two agency indicators were no longer statistically associated with recidivism. Additionally, the model indicated that individuals on probation were significantly more likely to recidivate than those sentenced to prison. Finally, after controlling for individuals’ risk and needs level, financial difficulties were no longer statistically associated with recidivism.

Although the LS/CMI criminogenic risk level provides valuable insight, it does not specify which criminogenic risk and needs factors are statistically associated with general recidivism in this sample. To address this, a third model was tested, incorporating all covariates from the first regression model along with the eight LS/CMI subscales. To enhance the interpretability of hazard ratios, the raw scores of these subscales were standardized. Seven of the eight risk and needs factors were significantly associated with recidivism, each in the expected direction. Among the eight LS/CMI subscales, only the antisocial pattern scale was not significantly associated with recidivism. The criminal history scale had the strongest association with recidivism, with a one-standard-deviation increase on that scale corresponding to a 47% higher risk of recidivism. The alcohol/drug problem scale showed the second strongest association, with a one-standard-deviation increase linked to a 24% higher risk of recidivism. The third model further indicated that the two agency indicators were not significantly related to recidivism. To further explore the importance of agency indicators, the third model was reanalyzed separately for each risk level (analysis not shown). In other words, the same model was retested but separately according to LS/CMI risk levels (i.e., very low, low, moderate, high and very high groups). Even after controlling for all covariates, neither agency indicator (motivation to change or denial/minimization of personal issues) was significantly associated with recidivism, regardless of risk and needs level (all *p*-values >.10).

### Examining the Interactions Effects

A series of Cox proportional hazard models were conducted to examine interaction effects between agency and criminogenic risk needs as well as with potential barriers (Table 3). All statistical models were adjusted for covariates, including offenders’ age, type of sentence, whether the index crime included a violent offense, and the number of adult convictions. For clarity, only the main effects of the two covariates and the interaction effect are reported. Interactions between agency and the eight criminogenic risk and needs factors were tested individually, followed by interactions between agency and each of the three potential barriers (homelessness, financial difficulties, and mental health problems). Each of these 11 interaction effects was tested separately to facilitate interpretation and prevent multicollinearity issues. The findings indicate that the absence of denial and minimization of personal issues was consistently associated with a significantly lower risk of recidivism across all tested models. Moreover, 9 of the 11 interaction effects involving this agency indicator were statistically significant (*p* < .001). For example, individuals who did not exhibit denial or minimization of personal issues (HR = 0.78) but had a score of one standard deviation above the mean on the LS/CMI criminal history subscale had a higher likelihood of recidivism (HR = 0.78 × 1.11 = 0.87). The same pattern was found for all criminogenic risk and needs, suggesting that such factors moderated, to some extent, the impact of agency. When examining barriers, only one of the three interaction effects was statistically significant. Financial difficulties moderated the effect of the absence of denial and minimization of personal issues on recidivism risk. Subsequently, the same interaction effects were tested using motivation to change as the agency indicator. All main effects were statistically significant, indicating that motivated individuals had a lower likelihood of recidivism. Furthermore, all 11 interaction effects tested were statistically significant (*p* < .001) and all in the same direction. In simple terms, motivated justice-involved individuals faced a higher likelihood of recidivism when a criminogenic risk/needs factor or a barrier was present. For instance, individuals who demonstrated motivation to change (HR = 0.61) but had a score one standard deviation above the mean on the LS/CMI alcohol/drug problems subscale had a significantly higher likelihood of recidivism (HR = 0.61 × 1.35 = 0.82) compared to those with lower scores on the subscale. In fact, among all criminogenic factors examined,^3^ alcohol/drug problems exhibited the strongest interaction effect.

**Table 3.** Interaction Effects Between the Absence of Denial/Minimization, Criminogenic Risk/Needs, and Potential Desistance Barriers on General Recidivism.

	Absence of denial and minimization			Presence of a motivation to change		
Covariates	Covariate (main effect)	Agency as absence of denial/minimization (main effect)	Covariate × agency (interaction effect)	Covariate (main effect)	Agency as presence of motivation to change (main effect)	Covariate × agency (interaction effect)
LS/CMI	Hazard ratio (Robust SE)	Hazard ratio (Robust SE)	Hazard ratio (Robust SE)	Hazard ratio (Robust SE)	Hazard ratio (Robust SE)	Hazard ratio (Robust SE)
Criminal history	2.12 (0.10)***	0.78 (0.04)***	1.11 (0.07)***	1.91 (0.11)***	0.65 (0.04)***	1.22 (0.08)***
Procriminal attitude	1.38 (0.05)***	0.85 (0.03)***	1.22 (0.07)***	1.17 (0.06)***	0.69 (0.04)***	1.32 (0.08)***
Antisocial pattern	1.75 (0.07)***	0.82 (0.04)***	1.16 (0.07)***	1.56 (0.08)***	0.71 (0.04)***	1.24 (0.08)***
Companions	1.53 (0.06)***	0.77 (0.03)***	1.17 (0.06)**	1.45 (0.07)***	0.70 (0.03)***	1.16 (0.07)***
Alcohol/drug problems	1.84 (0.07)***	0.70 (0.03)***	1.28 (0.07)***	1.67 (0.08)***	0.61 (0.03)***	1.35 (0.08)***
Leisure/recreation	1.59 (0.06)***	0.78 (0.04)***	1.13 (0.07)***	1.46 (0.08)***	0.69 (0.04)***	1.18 (0.07)***
Education/employment	1.74 (0.07)***	0.77 (0.04)***	1.10 (0.06)***	1.55 (0.08)***	0.65 (0.03)***	1.23 (0.08)***
Family/marital	1.51 (0.06)***	0.78 (0.04)***	1.11 (0.06)***	1.48 (0.07)***	0.71 (0.03)***	1.08 (0.06)***
Homelessness	1.30 (0.09)***	0.77 (0.02)***	1.18 (0.13)	1.13 (0.08)^†^	0.69 (0.02)***	1.49 (0.16)***
Mental health problems	1.29 (0.06)***	0.78 (0.02)***	1.07 (0.08)	1.18 (0.06)**	0.69 (0.02)***	1.22 (0.08)**
Financial difficulties	1.12 (0.04)**	0.72 (0.03)***	1.21 (0.07)***	1.07 (0.04)	0.64 (0.02)***	1.24 (0.07)***

*Note.* All models were conducted using a series of Cox proportional hazard models. All interaction effects were examined in separate models to avoid multicollinearity problems. All interaction effects were tested in separate regression models. All interaction terms were entered in a separate regression model other covariates are not shown. (a) The LS/CMI total was used as a covariate instead of the LS/CMI risk levels (b) The LS/CMI risk level was used as a continuous covariate.

NS = not statistically significant.

† *p* < .10. *p < .05. **p < .01. ***p < .001.

**Figure 4.**
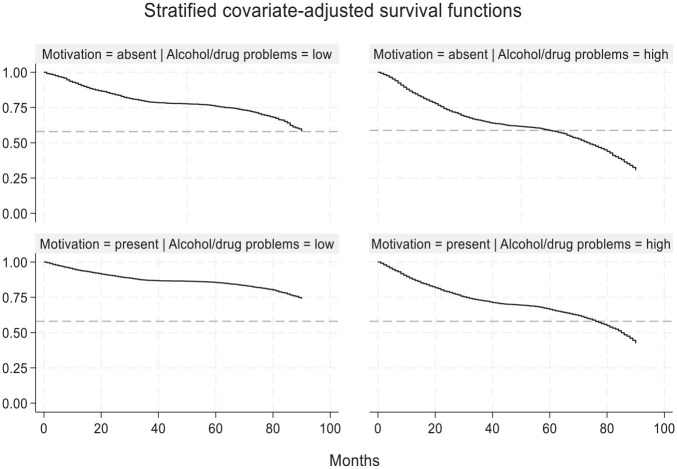
Adjusted survival function representing the interaction effects between agency (motivation to change) and criminogenic risk and needs (alcohol/drugs problems). *Note.* The Kaplan-Meier estimates are presented. The analysis present covariate-adjusted (age, sentence, index crime and number of adult convictions) survival curves. The LS/CMI subscale was dichotomized using a standard median-split. The horizontal line (dotted) refers to the base rate of general recidivism for this sample and is used only for comparative purposes.

This description, however, remains incomplete without examining the graphical representation of interaction effects. To visualize interaction effects involving LS/CMI criminogenic risk and needs (continuous variables), each subscale was dichotomized (i.e., median split, recoded as low/high) allowing to examine and compare survival curves. Figure 4 presents one of these significant interaction effects^4^ by comparing covariate-adjusted survival curves based on individuals’ scores on the agency indicator (i.e., motivation to change) and criminogenic risk and needs (i.e., LS/CMI alcohol/drug problems subscale). The horizontal dotted line represents the base rate of general recidivism (42% or 0.58 survival rate) for the entire sample, illustrating whether and how quickly each group reached this threshold. As expected, individuals who were motivated to change and had low criminogenic risk and needs exhibited the lowest probabilities of recidivism, never reaching the base rate threshold. Individuals who lacked motivation to change and had high criminogenic risk and needs displayed the highest recidivism rates, reaching the base rate threshold in just under 5 years. In contrast, individuals with high criminogenic risk and needs who were motivated to change had better outcomes than their unmotivated counterparts; recidivating at a lower rate and reaching the base rate threshold in 6½ years. Similar trends emerged when analyzing interaction effects involving barriers. These findings suggest that, regardless of criminogenic risk and needs or the presence of barriers, motivated individuals tend to fare better in the community than unmotivated individuals.

## Discussion

Criminogenic risk and needs play a critical role in justice-involved individuals’ decisions to reoffend, and the findings of the current study provide additional evidence supporting this. Although this study did not assess the predictive validity of the LS/CMI, its findings align with existing research, demonstrating that criminogenic risk and need factors, as measured by the LS/CMI, offer insight into recidivism probabilities. The risk probabilities increased steadily in accordance with the assigned criminogenic risk and needs level determined by the LS/CMI. For instance, individuals classified as very high in criminogenic risk and needs were 13 times more likely to experience a recidivism event during the follow-up period compared to the very low-risk group. These findings highlight the relatively stable nature of recidivism risk, but it does not speak about the dynamic aspect of it. While dynamic criminogenic risk and needs were measured, their stability and evolution were not. The extent to which these factors changed during the study period remains unclear. These changes may or may not occur during an individual’s sentence, complicating the assessment of criminogenic risk and needs over time.^5^ Researchers have long identified various dynamic risk factors (e.g., Andrews et al., 2006; Douglas & Skeem, 2005; Zamble & Quinsey, 2001) and their statistical association with recidivism (e.g., Caudy et al., 2013). An ongoing challenge, however, has been determining whether intraindividual changes meaningfully reflect shifts in recidivism risk (e.g., Davies et al., 2023; Kroner & Yessine, 2013). Research on intraindividual changes in dynamic risk factors suggests that these shifts correlate with changes in offending over time (e.g., E. McCuish et al., 2020; Rocque et al., 2016) but more work is needed to understand the process of change and the role of agency in this process.

It is well recognized that recidivism is a relatively poor indicator of change (e.g., Klingele, 2019; Lussier & Cale, 2013). The criminological literature on recidivism has yet to produce a theoretical model of change while accounting from individuals’ levels of criminogenic risk and needs (see Horan et al., 2020). Developing such a model of change would require recognizing the role of agency, not as the sole determinant of desistance, but as an integral part of the equation. In the context of the study, self-determination was measured by an individual’s ability to acknowledge personal issues and their expressed motivation to change. Drawing from Prochaska and Velicer (1997) transtheoretical model of change, both indicators used in this study may correspond to different stages of change. Recognizing personal issues suggests a precontemplation stage, where an individual considers the possibility of change. In contrast, expressing motivation to change may indicate contemplation, signaling an intention to take action. The second indicator, motivation to change, may more directly reflect agentic decision-making, signaling an individual’s commitment to making life choices conducive to desistance from crime. After statistically controlling for individuals’ level of risk as measured by the LS/CMI, agency related indicators were no longer statistically related to recidivism highlighting the importance of criminogenic risk and needs to prevent recidivism. Our study showed that recognizing personal issues and expressing a motivation to change were significant moderators of criminogenic risk and needs, including what are considered “static risk factors” (i.e., criminal history indicators). For some individuals, including those with extensive criminal histories, recognizing personal issues and expressing motivation to change can signal a turning point in the life course. However, this study did not examine the specific actions individuals took to actively influence their own life trajectories, the effectiveness of such actions, or whether they perceived the consequences of these actions as positive.

The importance of integrating research from RNR and desistance is highlighted by the interaction effects found between criminogenic risk and needs and agency. Simply ignoring criminogenic risk and needs in favor of agency is not supported by the study findings. More specifically, agentic decisions and actions, such as recognizing personal issues and demonstrating motivation for personal changes matter especially when individuals are presenting high criminogenic risk and needs. On the one hand, such motivation may not be a necessary condition for individuals who are already low in terms of their criminogenic risk and needs and who are unlikely to reoffend. On the other hand, our study also suggests that, transitioning to a crime-free lifestyle can be particularly challenging for individuals with high criminogenic risk and needs (Healy, 2017; Nugent & Schinkel, 2016). Notably, the study showed that individuals with high and very high criminogenic risk and needs are 9 to 13 times more likely to re-enter the criminal justice system in a context when RNR principles are in place. For most, this return occurs within 1 or 2 years, possibly highlighting the shortcomings of interventions and/or the context surrounding those. While many justice-involved individuals expressed a motivation to change, this suggests that criminal justice professionals should consider integrating desistance-focused interventions (e.g., Farrall & Maruna, 2004) to RNR principles. Such interventions can capitalize on agency and self-determination, but also these individuals’ personal and social strengths and resources (e.g., K. J. Fox, 2022).

It remains unclear how criminal justice professionals take justice-involved individuals’ motivation to change into account in the context of high criminogenic risk and needs and the associated risk of recidivism. Desistance research identified key principles that could inform criminal justice practices in that context. These include recognizing the importance of desistance as a subjective process rather than applying a one-size-fits-all approach, working collaboratively with individuals rather than imposing change upon them, and fostering a positive relationship (e.g., Ainslie, 2021; McNeill & Weaver, 2010; Weaver & McNeill, 2010). The importance of integrating desistance-focused principles with RNR principles of intervention is further highlighted by the current study findings which showed that, contrary to what has been observed in prior studies (Villettaz et al., 2015), probation was associated with higher recidivism rates after criminogenic and needs were statistically controlled for. This could reflect a number of factors, such as strict conditions imposed to probationers by the court, the poor quality of probation services, the heavy probation caseloads, the use of ineffective intervention skills, as well as the lack of time and adequate resources to intervene on criminogenic risk and needs (e.g., Bonta et al., 2011; C. Fox et al., 2022; Mitchell et al., 2024). These findings underscore the need to examine, among other things, how probation officers perceive and integrate agency into their assessment and interventions for individuals in the community (e.g., King, 2013b).

While the current study examined individuals’ recognition of personal issues and their openness to change, it did not include other key theoretical components of desistance. While these two agentic factors are associated with early stages of change, other factors are considered crucial in later stages of desistance. Theories of desistance differ on the key factors influencing the process of desistance (Cid & Martí, 2017), but cognitive transformation theory (P. C. Giordano et al., 2002) posits that beyond openness to change, factors such as access to hooks for change, identity change or how one sees oneself, as well as changes as to the meaning and desirability of antisocial and criminal behavior are other critical components of transformation (see also, Giordano, 2022). Further exploration of cognitive transformation theory should assess whether interventions targeting criminogenic needs facilitate this transformation process. Maruna and Mann (2019) proposed the concept of “justice capital” to emphasize the importance of equitable access to social, physical, human, and cultural resources in enhance criminal justice interventions. As suggested in this study, such access remains a challenge for justice-involved individuals, especially those experiencing mental health problems, financial struggles, and homelessness. These findings highlight that such challenges and structural barriers, some of which falls outside the scope of the criminal justice system, can hinder a person’s ability to desist from crime, even when they are motivated to do so. Homelessness, mental health problems and financial struggles stem from broader structural and systemic issues that fall beyond the realm of criminal justice policies and interventions. Difficulties finding a place to live due to the housing/rental market, the lack of access to mental health services in the community, delays and costs associated with such services, as well as the economic reality that may impact a person ability to meet ends all underscore the necessity of multi-ministerial involvement and inter-agency collaboration to support desistance efforts collectively.

### Study Limitations

The current study is not without limitations. As the study consists almost exclusively of French Canadians, cultural differences may limit the generalizability of its findings elsewhere. Although the sample consists of individuals under the jurisdiction of a provincial correctional services, approximately half of the sample was classified as high or very high risk according to the LS/CMI. Even if this sample was serving relatively short sentences, this was by no means a low-risk group; on average, this sample had close to seven prior convictions. Although the study is concerned with the dynamic nature of human lives, it lacks repeated measurements of criminogenic risk and needs, preventing an assessment of change over time. Additionally, crude measures of agency were available for these large-scale study, as more detailed and precise indicators were not available. No measure of social desirability was included in the study which might have influenced how some justice-involved individuals presented themselves to criminal justice officials. It is important to emphasize that agency was measured by criminal justice professionals while justice-involved individuals were serving their sentences. Despite their training to conduct such assessments, this context may have influenced how agency-related indicators were measured. The measurement of recidivism was limited to official sources of data as a criterion for determining that recidivism occurred during the follow-up period. The follow-up period was about 5 years on average and some individuals who appeared as non-recidivist in the current study might be classified as recidivism with a longer follow-up period. This limitation is not critical given that (a) the yearly failure rate significantly dropped after the first 1 to 2 years of the follow-up period; (b) surviving longer without a recidivism event remains consistent with the process of desistance which refers to a slowing down of offending over time.

## Conclusion

Despite its challenges and limitations, the RNR model for service delivery in correctional settings has been supported by empirical evidence demonstrating its value and relevance. However, research has largely overlooked the third component of this model, individuals’ responsivity to interventions, which offer possibilities for theoretical integration with desistance research and the concept of agency. Notably, the RNR model does not include a framework describing the process of change that leads to the termination of criminal behavior. In contrast, desistance research highlighted agency as a crucial factor in this process, at least for some justice-involved individuals. Findings from this study, based on 14,000 adults followed in the community for an average of 5 years, suggest that agency alone is not sufficient for some individuals, particularly those facing challenging life adversities. Agency, however, can complement RNR principles. Specifically, recognizing personal issues and expressing motivation to change may signal a turning point in an individual’s life course. Our findings suggest that agency can moderate, to some extent, criminogenic risk and needs, reinforcing the importance of correctional-based programs that support agentic decisions and actions toward desistance from crime. However, while recognizing personal issues and demonstrating motivation to change are important, they do not guarantee immediate behavioral change, particularly in terms of recidivism. Although these agentic factors may mitigate some criminogenic risks and needs, their impact is constrained by barriers such as mental health issues, homelessness, and financial struggles. In the face of such life adversities, internal motivation alone may be insufficient as structural and systemic interventions that extend beyond the scope of the criminal justice system warrant closer attention. This is particularly critical for individuals facing mental health challenges, economic hardship, and housing instability, as these factors can undermine agency and contribute to further marginalization while increasing the likelihood of reoffending.

## Notes

1. In the province of Quebec, “criminologists” are generally university-trained criminal justice professionals with a bachelor’s degree in criminology whose training is specifically oriented toward tasks such as the assessment and individual-based and group-based interventions with justice-involved individuals.

2. It would be redundant to incorporate diagnostic of an antisocial personality disorder (APD) given that the LS/CMI’s C8 factors tap into several aspects of the APD (e.g., antisocial attitudes).

3. The same analyses were conducted using the LS/CMI risk level as a covariate to examine interaction effects with the two agency indicators. The findings were aligned with those reported for the eight LS/CMI subscale. Interaction effects between the LS/CMI risk level and the measure of denial/minimization (HR = 1.08; 95% CI [1.02, 1.15]) as well as with the measure of motivation to change (HR = 1.10; 95% CI [1.03, 1.18]) were both statistically significant (*p* < .01).

4. Only one interaction effect is graphically presented due to space limitation.

5. Because the LS/CMI is used by criminal justice professionals to guide service delivery, determine the intensity of such interventions, and inform community supervision, assessing the predictive accuracy of any risk and needs instrument is difficult. More specifically, effective correctional-based and community supervision interventions may have moderated the impact of risk and need factors, a possibility that could not be assessed in this study.
